# Effects of Polyol Types on Underwater Curing Properties of Polyurethane

**DOI:** 10.3390/polym17010005

**Published:** 2024-12-24

**Authors:** Cheng Zhang, Yixuan Zhang, Yao Liu, Yiming Cui, Ming Zhao, Shuai Peng, Hecong Wang, Zuobao Song, Qunchao Zhang, Dean Shi, Yuxue Zhu

**Affiliations:** 1 State Grid Beijing Power Cable Company, Beijing 100022, China; zhangchengpower@163.com (C.Z.); phxsuns924@163.com (Y.Z.); 15727362608@163.com (Y.L.); cym828@126.com (Y.C.); zhaoming1989@hotmail.com (M.Z.); 2 CNBM Zhongyan Technology Co., Ltd., Beijing 100024, China; shuaipeng2019@163.com (S.P.); whc13833987118@163.com (H.W.); 3 China Building Materials Academy Co., Ltd., Beijing 100024, China; songzuobao@cbma.com.cn; 4 School of Materials Science and Engineering, Hubei University, Wuhan 430062, China; zhangqc1976@hubu.edu.cn (Q.Z.); deanshi2012@hubu.edu.cn (D.S.)

**Keywords:** castable polyurethane, bio-based polyols, underwater construction, curing properties

## Abstract

This study aims to develop castable polyurethane suitable for applications on wet substrates or underwater construction. Polyurethanes were synthesized using various polyols with similar hydroxyl values, including poly(tetrahydrofuran) polyol, polyester polyol, castor oil-modified polyol, soybean oil-modified polyol, and cashew nut shell oil-modified polyol. The corresponding polyurethane curing products were evaluated for their underwater curing characteristics by volume expansion ratios and adhesion strength on dry and wet substrates, combined with analyses of reaction exothermic behavior, wetting properties on dry and wet substrates, interfacial tension, and microstructure characterization from the perspectives of reaction activity and water solubility. The results indicate that polyols with higher hydrophobicity and reactivity to isocyanates lead to reduced side reactions during underwater curing, making them more suitable for underwater applications. Soybean oil-based and cashew nut shell oil-based polyurethanes exhibited fast curing (gel times of 1.15 and 1.35 min, respectively), minimal volume change (within 2.5% after 7 days underwater), and strong wet adhesion (1.95 MPa and 2.38 MPa with minimal loss, respectively). The two polyols showed different mechanical properties, providing tailored options for specific underwater engineering applications.

## 1. Introduction

Polyurethane is a polymer formed by the reaction of isocyanates with hydroxyl groups, resulting in urethane linkage [1]. Compared to traditional cement-based materials, polyurethane offers advantages such as rapid reaction times, customizable mechanical properties, and excellent injectability [2]. These properties make polyurethane widely used in various construction fields, including railways [3,4], roads and bridges [5,6,7,8], water conservancy [9,10,11,12], and mining [13,14].

Due to the complex and diverse nature of real-world engineering environments, polyurethane is often required for applications on wet substrates or underwater conditions. However, isocyanate groups are susceptible to side reactions with water, leading to the release of carbon dioxide and the formation of polyurea [15]. This alters the polyurethane’s cured structure, reducing its mechanical properties and compromising the overall project quality. To mitigate the side reactions between water and isocyanates and to avoid issues such as performance degradation and dimensional instability, researchers have focused on two main optimization strategies: modifying the hydrophobic components of the isocyanates or polyols, and adjusting the catalytic reaction rates. Furthermore, the impact of curing underwater conditions on polyurethane properties has been explored. Hannay [16] introduced a formulation for foamed polyurethane that incorporates non-volatile, water-immiscible components (e.g., hydrocarbons) to suppress the side reactions between isocyanates and water, thereby addressing the impact of groundwater on the foaming performance of polyurethane. Botrie et al. [17] developed hydrophobic, closed-cell polyurethane for use in load-bearing piles, incorporating water-immiscible components such as crude oil, tar oil, or vegetable oils and selecting hydrophobic hydrocarbons, polybutadiene polyols, and bio-based polyols as reactive hydrogen sources. Stamenkovic [18] explored the catalytic effects of various metal catalysts on the reaction rates of isocyanates with hydroxyl groups and water, using Fourier transform infrared spectroscopy (FTIR). Li et al. [19] studied a polyurethane-epoxy resin composite for grouting and repair in soft soil foundations, groundwater-rich areas, and sites with water inrush or slurry flow. The results indicated that the curing time extended under underwater conditions and the inclusion of polyurethane effectively improved wet substrate adhesion. Li et al. [20] developed polyurethane foam materials for underwater applications and investigated the effect of underwater curing and water immersion on the foam structure and mechanical properties. Prisacariu et al. [21] examined the curing mechanism of cast polyurethane, highlighting the influence of water molecules on the curing process and reaction kinetics. Yuan et al. [22] simulated the process of grout injection into water under varying proportions and flow rates, quantitatively testing the erosion resistance of polyurethane grout under water flushing and dilution, showing that polyurethane grout exhibited superior erosion resistance compared to cement-based grout. Hao et al. [23] investigated the diffusion behavior of polyurethane grout in water-bearing fractures, focusing on the effects of grouting quantity and hydrostatic pressure on diffusion characteristics. Overall, while substantial research has been conducted on the macroscopic effects of water on the structure and performance of polyurethane, studies focusing on the microscopic scale and curing mechanisms are relatively limited.

This study focuses on the effects of curing properties and microstructure on macroscopic underwater curing behavior and mechanical properties. Five commercially available polyols—polyether polyol, polyester polyol, castor oil-modified polyol, soybean oil-modified polyol, and cashew nut shell oil-modified polyol—were selected based on their similar hydroxyl values and water-immiscibility. These polyols were reacted with the same isocyanate component to synthesize polyurethane. Using techniques such as interfacial tension measurement and microstructural analysis, this study investigates the effect of polyol type on the underwater curing behavior and macroscopic mechanical properties of polyurethane. The results provide valuable evaluation criteria and guidance for applying polyurethane materials in water-containing environments.

## 2. Materials and Methods

### 2.1. Materials

PM200 (polyaryl polymethylene isocyanate, PAPI) was provided by Wanhua Chemical Group Co., Ltd., Yantai, China. The terminal isocyanate-based polyurethane prepolymer was prepared in-house. PTMG650 (polyether polyol, PTMG) was supplied by BASF Shanghai, China. 22,170 (polyester polyol, PES) was obtained from Zhejiang Huafon Synthetic Resin Co., Ltd., Zhejiang, China. H368 (castor oil-modified polyol, COP) was supplied by Ito Oil Chemicals Co., Ltd., Shanghai, China, while 13,180 (soybean oil-modified polyol, SOP) was purchased from Guangzhou Haierma Vegetable Oils and Fats Co., Ltd., Guangzhou, China. 9001LV (cashew nut shell oil-modified polyol, CNSOP) was sourced from Cardolite Chemical Zhuhai Co., Ltd., Zhuhai, China. 1,4-Butanediol (BDO) was supplied by Shanghai Macklin Biochemical Technology Co., Ltd., Shanghai, China. Organometallic catalyst LK380 was obtained from Newtop Chemical Materials (Shanghai) Co., Ltd., Shanghai, China. Solvent oil D80 was provided by Guangdong Zhonghai Nanlian Energy Co., Ltd., Maoming, China. All polyols and 1,4-butanediol were vacuum-dried at 120 °C for 2 h prior to use.

### 2.2. Preparation of Polyurethanes

**Figure 1:**
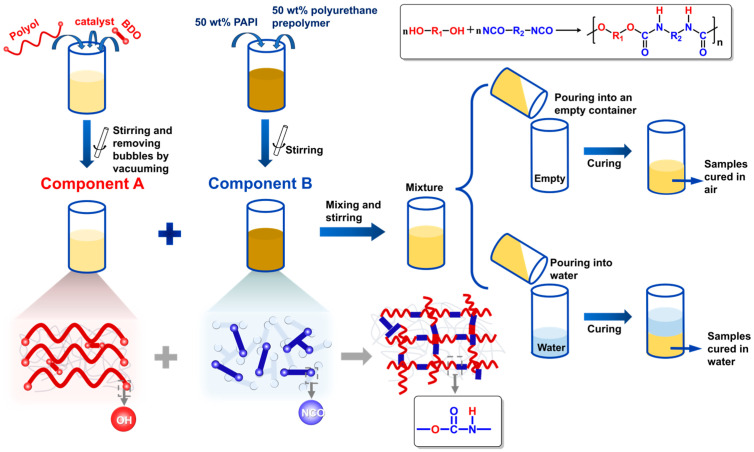
Sample preparation process.

To better simulate the curing behavior of polyurethanes in a water-containing environment, this experiment was conducted under standard room conditions (23 °C, 50% relative humidity) without external heating. The preparation process for the samples is illustrated in Figure 1.

The required quantities of polyols, 1,4-butanediol, and catalyst (10 wt% organometallic catalyst in D80 solution) were mixed thoroughly and subjected to vacuum drying at 50 °C for 15 min, followed by cooling to room temperature, and are designated as Component A. PAPI and a self-prepared isocyanate component were blended at a 1:1 mass ratio to form the isocyanate component, designated as Component B. Components A and B were then weighed to achieve a total mass of 100 g, thoroughly mixed by stirring for 10 s, and subsequently evenly distributed into an empty container and a container containing 5 cm of water in order to simulate the process of slurry mixing in equipment such as grouting machines and being injected through water into the filling area. The specific mass ratios for each sample are listed in Table 1.

### 2.3. Measurements

#### 2.3.1. Fourier Transform Infrared (FTIR)

FTIR spectra were recorded using a TENSOR 37 spectrometer (Bruker, Karlsruhe, Germany), employing KBr crystal windows. Each sample was scanned for 32 times over a wavenumber range of 4000–400 cm^−1^.

#### 2.3.2. Reaction Process Temperature Monitoring

The reaction temperature was monitored using a TA612C thermocouple thermometer (TASI, Suzhou, China). Components A and B (total mass of 100 g) were mixed for 10 s before inserting the temperature probe 3 cm below the mixture surface, and temperature variations during the reaction process were recorded.

#### 2.3.3. Gel Time

The gel time was measured using an NJ480s rotational viscometer (Shjingmi, Shanghai, China), following the GB/T 7193-2008 standard [24]. Measurements for each sample were performed in duplicate.

#### 2.3.4. Spreading Diameter and Interfacial Tension Measurement

Spreading diameter and interfacial tension were performed using a DataPhysics OCA 15EC optical contact angle measuring instrument (DataPhysics, Filderstadt, Germany). The spreading diameter measurements employed the sessile drop method, with dry and wet substrates prepared in accordance with GB/T 16777-2008, Chapter 6 [25]. Interfacial tension was measured using the pendant drop method, with three tests conducted per sample and the median value recorded.

#### 2.3.5. Volume Expansion Ratio

The volume expansion ratio was determined using an FK-120DT density meter (FURBS, Xiamen, China). Under standard temperature and humidity, two 50 g portions of the mixed slurry were poured into containers: one in air and one submerged in 5 cm of water. After curing for 1 and 7 days, the density of the cured product was measured with the density meter. The volume expansion ratio was calculated using Equation (1):

(1) $$\delta =\frac{V_{1}-V_{0}}{V_{0}}\times 100\%=\frac{\rho_{0}}{\rho_{1}}-1$$

where

*δ* = volume expansion ratio (%);

*V*_1_ = volume of the cured product (cm^3^);

*V*_0_ = volume of the slurry (cm^3^);

*ρ*_0_ = density of the slurry (g/cm^3^);

*ρ*_1_ = density of the cured product (g/cm^3^).

#### 2.3.6. Scanning Electron Microscopy (SEM)

The morphology of the samples was characterized using a FEI Quanta FEG250 field emission scanning electron microscope (FEI, Hillsboro, OR, USA). Samples were gold-coated in a vacuum coater for 3 min and then attached to conductive tape prior to testing. Analysis was conducted at an acceleration voltage of 10 kV with magnifications of 200× and 2000×.

#### 2.3.7. Adhesion Properties

Adhesion tests were carried out using a UTM4104X universal testing machine (Sunstest, Shenzhen, China) in accordance with the JC/T 1041-2007 standard [26], with a test speed of 5 mm/min.

#### 2.3.8. Mechanical Properties

**Figure 2:**
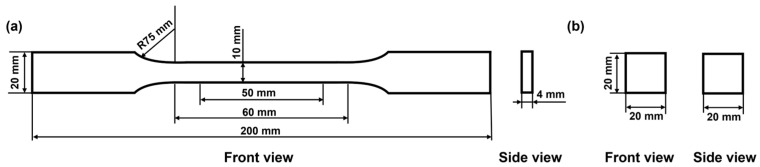
Specimens for mechanical tests. (**a**) Specimens for tensile test; (**b**) specimens for compression test.

The components A and B were thoroughly mixed for 10 s, poured into silicone molds of specified dimensions, and cured at 23 °C and 50% relative humidity for the required duration before testing. The dimensions of the specimens are shown in Figure 2.

Tensile tests were conducted using a UTM4104X universal testing machine (Sunstest, Shenzhen, China) according to the GB/T 2567-2008 standard [27], with a test speed of 10 mm/min.

Compressive strength was evaluated using a TestPilot E10C universal testing machine (Wance, Shenzhen, China) following the GB/T 2567-2008 standard. The sample dimensions were 2 × 2 × 2 cm^3^, and the test speed was set at 5 mm/min. The compressive strength was taken as the maximum value within the yield or 15% strain range.

## 3. Results

### 3.1. Effect of Polyol Types on Underwater Curing Reaction Characteristics of Polyurethane

#### 3.1.1. Structural Characterization of Polyurethane

**Figure 3:**
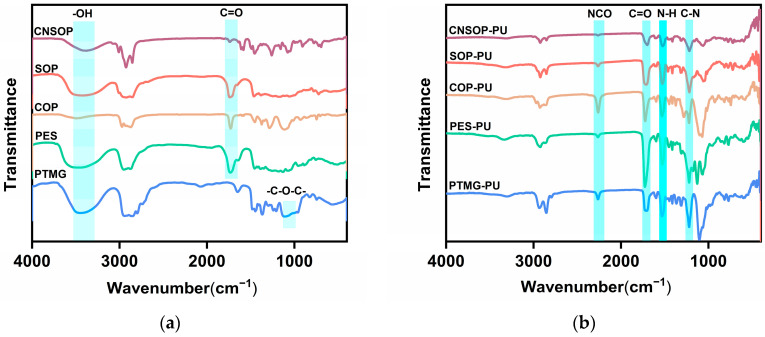
FTIR spectra of polyols before and after the reaction. (**a**) FTIR spectra of polyols; (**b**) FTIR spectra of the corresponding polyurethanes derived from the polyols.

Figure 3 presents the FTIR spectra before and after the reaction of polyols. As shown in Figure 3a, a broad absorption peak in the range of 3500~3250 cm^−1^ corresponds to the stretching vibration of the –OH group, indicating the presence of hydroxyl functional groups in the molecular structure of the sample, as reported in the relevant literature [28,29,30,31]. Notably, PTMG exhibits a strong, broad absorption peak around 1107 cm^−1^, which is attributed to the stretching vibration of the C–O–C bond in aliphatic ether linkages, a characteristic feature of polyether polyol structures. For other polyols, an absorption peak in the range of 1750~1700 cm^−1^ is observed, corresponding to the stretching vibration of the ester carbonyl group (C=O). This suggests the presence of ester linkages, indicative of polyester polyols and bio-based polyols. After the reaction with isocyanates, Figure 3b shows the disappearance of the hydroxyl absorption peak at 3400 cm^−1^ and the emergence of a new peak around 3300 cm^−1^, which is attributed to the N–H stretching vibration of the urethane group (–NH–COO–). This shift indicates that the hydroxyl groups have been consumed, leading to the formation of urethane linkages. Additionally, a strong C=O absorption peak appears in the range of 1750~1730 cm^−1^, characteristic of the amide I band, while peaks between 1520 and 1560 cm^−1^ correspond to N–H bending vibrations (amide II), and a peak at 1240~1220 cm^−1^ is associated with C–N stretching (amide III). These features confirm the successful formation of the polyurethane structure. An absorption peak at 2260 cm^−1^, corresponding to the antisymmetric stretching vibration of the –NCO group, indicates the presence of unreacted isocyanate groups in the system. This peak is most pronounced in PTMG-PU and COP-PU, suggesting a slower reaction rate for these samples, which is consistent with results from subsequent analyses.

#### 3.1.2. Study on the Exothermic Process of Curing Reactions

The rate of the side reaction between isocyanates and water is influenced by several factors, including the structure of the isocyanate, the type of catalyst, the concentration of water molecules, and the temperature of the reaction system. In this study, during polyurethane curing at ambient temperature, the temperature variation within the system is primarily attributed to the heat released by the reaction between the polyol components and the isocyanate groups. This temperature change not only reflects the reaction rate between the polyol and isocyanate but also further accelerates the progression of the reaction. Regarding the side reaction between isocyanates and water, it is theoretically understood that lower temperatures reduce the rate of this side reaction, thereby minimizing its occurrence. However, lower temperatures also slow the consumption of isocyanate groups by the polyol components. Consequently, the prolonged exposure of isocyanate groups to water molecules may, in turn, facilitate the side reaction. To explore the combined effects of temperature on the side reaction and the curing performance in the presence of water, this study monitors the temperature variations throughout the reaction process for five different sample groups. Additionally, the gel times for each system are compared to assess the relationship between system temperature and the extent of the side reaction during curing.

**Figure 4:**
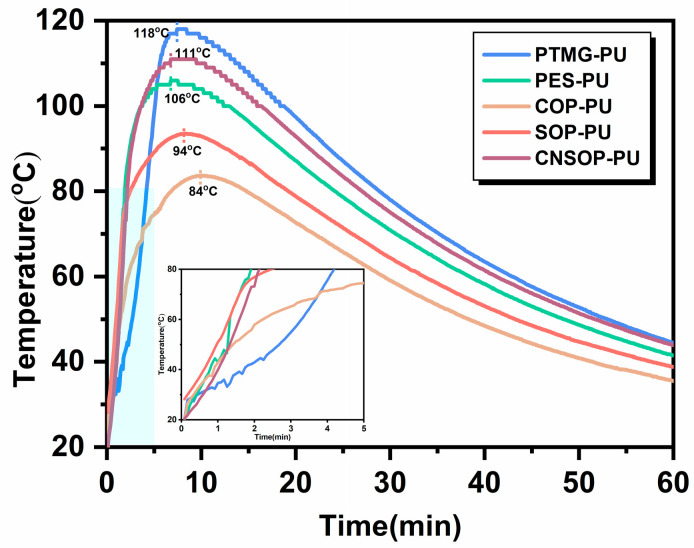
Temperature–time curve of five reaction systems.

**Figure 5:**
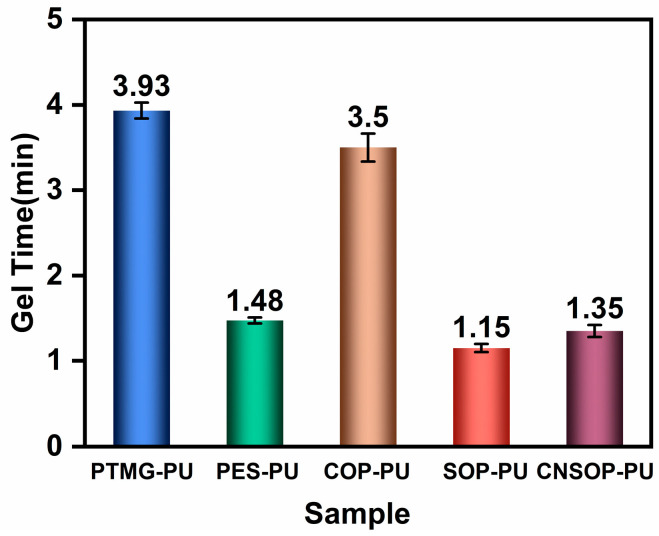
Gel time of the reaction systems.

Figure 4 illustrates the variation in system temperature over time for the samples, with a rapid initial temperature increase followed by a gradual decrease. Figure 5 presents the actual gel times of the samples. Under identical conditions for isocyanate components and catalyst concentration, PTMG-PU exhibits the highest peak reaction temperature, reaching 118 °C at 7.4 min. PES-PU attains a maximum temperature of 106 °C at 6.75 min. These higher temperatures can be attributed to the greater reactivity of primary hydroxyl groups compared to secondary ones. For PTMG-PU and PES-PU, component A has an average functionality of 2, resulting in the longer gel time for PTMG-PU of approximately 4 min. In contrast, the ester groups in the polyester polyol have higher polarity than the ether bonds in the polyether polyol, leading to stronger hydrogen bonding interactions between the polyester polyol’s soft segments and the hard segments formed by isocyanates and chain extenders. These stronger interactions promote physical crosslinking, reducing the gel time to 1.48 min for PES-PU. For the bio-based polyurethane samples, COP-PU, which uses castor oil-modified polyol, shows the lowest overall reaction temperature, peaking at only 84 °C, with a gel time of 3.5 min. The slower reaction rate can be attributed to the secondary hydroxyl groups in the castor oil polyol, which are less reactive toward isocyanates compared to primary hydroxyl groups. Additionally, COP-PU has the lowest functionality (approximately 2.5) among the bio-based polyols, further contributing to the slower reaction rate. SOP-PU and CNSOP-PU, which use soybean oil-modified polyol and cashew nut shell oil-modified polyol, respectively, exhibit similar initial reaction rates and gel times. However, CNSOP-PU has a higher functionality, and, given that both samples have comparable hydroxyl values, the higher functionality in CNSOP-PU leads to a faster reaction rate. Consequently, the system temperature for CNSOP-PU is higher, reaching 110 °C.

**Figure 6:**
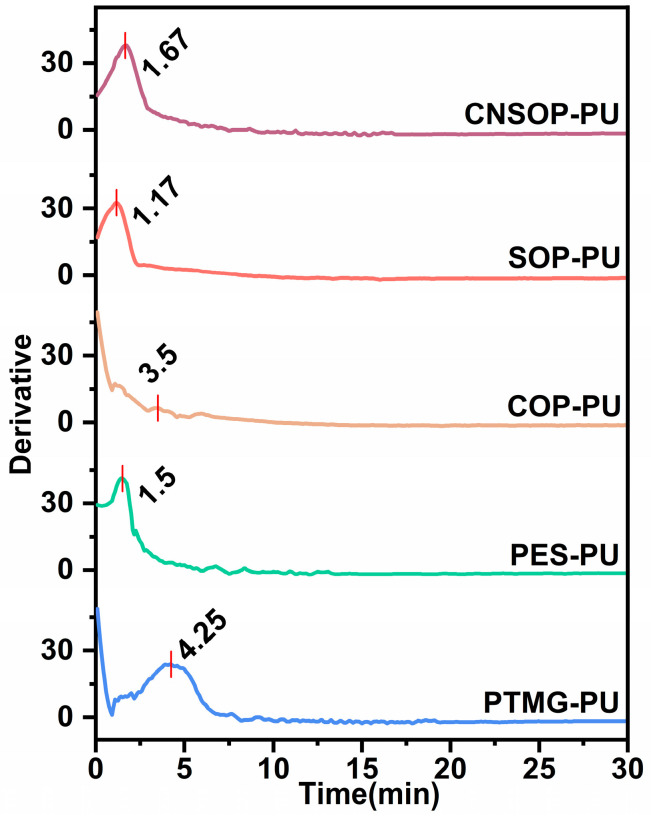
Rate of temperature change over time for different samples.

To further explore the relationship between curing behavior and system temperature, the derivative of the temperature–time curve shown in Figure 4 was calculated, yielding Figure 6. As shown in the graph, the peak values of the differential curves closely align with their respective gel times. Specifically, the gel point for each sample corresponds to the maximum temperature change within the given time interval. At this stage, the hydroxyl groups and isocyanates undergo progressive polymerization, leading to crosslinking within the system. This results in a sharp increase in viscosity, making the system more resistant to flow. Consequently, the material transitions into a gel-like state, hindering the rise of internal bubbles.

#### 3.1.3. Macroscopic State Comparison of Underwater Curing

**Figure 7:**
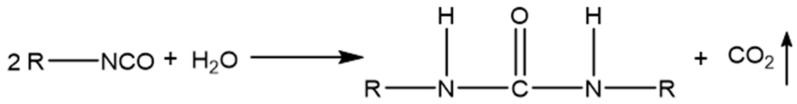
Reaction formula of isocyanates with water [1].

The reaction between isocyanates and water, as shown in Figure 7, produces carbon dioxide gas, which is a primary factor contributing to the reduced mechanical properties and poor dimensional stability of polyurethanes in underwater environments. This section investigates the volume expansion ratios of the samples cured and exposed to both air and water, quantifying the dimensional stability of polyurethanes synthesized from various polyols during underwater curing. These measurements also serve as an indirect indicator of the extent of side reactions.

**Figure 8:**
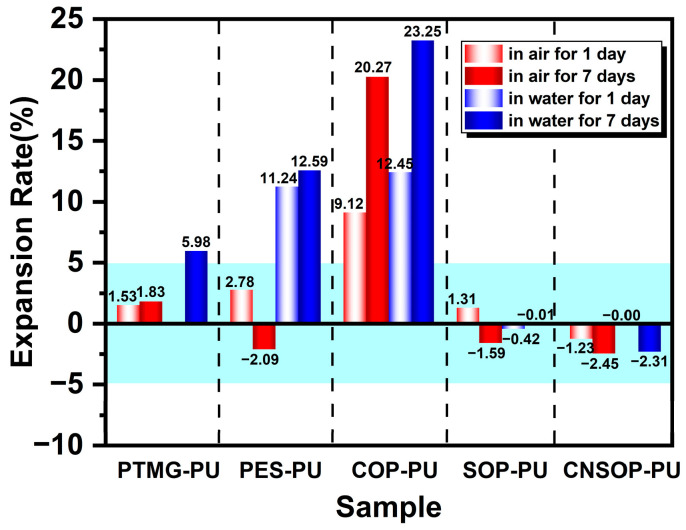
Volume expansion ratio of samples cured in air and water.

**Figure 9:**
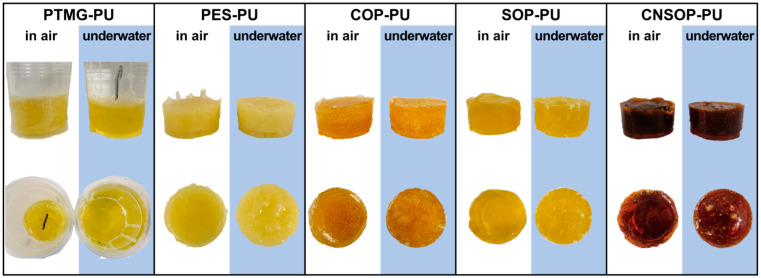
Photographic comparison of samples cured in air and water.

Figure 8 shows the volume expansion ratios of the cured products over different curing times, while Figure 9 presents a visual comparison of the samples cured in air and underwater for one day. PTMG-PU, which did not fully react, could not be extracted, and bubble traces were visible along the container walls. After 7 days, this sample exhibited a volume expansion ratio of 5.98%. Due to its slow reaction rate and low crosslinking density, internal bubbles were able to escape through the surface, resulting in a measured volume expansion ratio lower than the theoretical value. PES-PU showed a significant increase in volume expansion when cured underwater. After 1 day in water, the expansion ratio increased from 2.78% in air to 11.24%. This substantial increase can be attributed to the poor hydrophobicity of the polyester polyol, suggesting that this sample is unsuitable for underwater curing conditions. COP-PU exhibited numerous internal bubbles in both air and water, with the highest volume expansion ratios observed: 9.12% in air and 12.45% underwater after 1 day. Based on the reaction temperature–time profile (Figure 4), it is inferred that the low reactivity of the castor oil-modified polyol led to an extended gel time, slowing the consumption of isocyanate by active hydrogen components. This extended reaction time allowed more isocyanate groups to react with water molecules. Additionally, the polyol’s high functionality and crosslinking density also caused a rapid increase in viscosity, trapping more bubbles within the system, resulting in the highest volume expansion ratio in COP-PU. In contrast, SOP-PU and CNSOP-PU, which were synthesized with polyols of higher functionality, faster reaction rates, and hydrophobic long alkyl chains, showed better performance under underwater curing conditions. Only minor surface voids were observed, and the overall volume changes exhibited a shrinking trend. This contraction is likely due to the transition from van der Waals forces to covalent bonding during curing, as covalent bonds are shorter than van der Waals forces, allowing polymer chains to pack more tightly, leading to volume reduction [32]. The volume change for both samples was within a 2.5% range. In summary, considering the volume expansion ratios, the soy oil-based polyurethane (SOP-PU) and cashew nut shell oil-based polyurethane (CNSOP-PU) are more suitable for underwater curing applications.

#### 3.1.4. Microscopic Morphology Comparison of Underwater Curing

**Figure 10:**
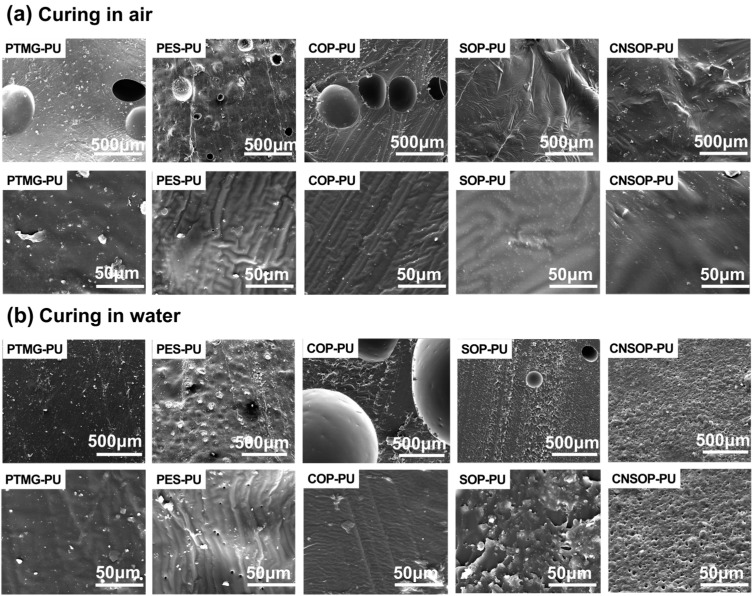
Microscopic morphology comparison of samples cured in air and water. (**a**) curing in air; (**b**) curing in water.

To further investigate the effects of underwater curing on the microstructure of the products, Figure 10 presents scanning electron microscope (SEM) images of the cross-sections of each sample. In PTMG-PU, the cured product in air displays larger through-holes and flat depressions, likely caused by air bubbles introduced during stirring or pouring, as well as carbon dioxide produced by side reactions. Upon higher magnification, the structures appear dense, and no small voids with aggregated hard segments structures are observed. The underwater-cured sample exhibits a similar structure, with hard segments dispersed within soft segments. Since the pores are concentrated on the surface (as shown in Figure 9), no bubbles are observed in the cross-sectional area. PES-PU exhibits visible pores in both air- and underwater-cured products, with the underwater-cured sample showing an increase in pore number and size. This suggests that side reactions are more pronounced under underwater conditions, aligning with the increased volume expansion ratio shown in Figure 8. COP-PU features smooth pore edges, a flat fracture surface, and regularly arranged striations, indicating brittle fracture behavior. This sample has the largest pore sizes, which further increase in the underwater-cured product, consistent with its high volume expansion ratio. This further supports the idea that side reactions lead to a reduction in the mechanical properties. In the underwater-cured products of SOP-PU and CNSOP-PU, pores are also observed; however, both the number and the size of pores are significantly smaller compared to the first three samples, explaining the less pronounced volume expansion observed. In summary, underwater curing impacts the microstructure of the samples, generally increasing either the number or size of pores. However, samples with smaller macroscopic volume changes tend to exhibit fewer or smaller pores.

#### 3.1.5. Effect of Different Polyol Types on Adhesion Strength on Wet Surfaces

**Figure 11:**
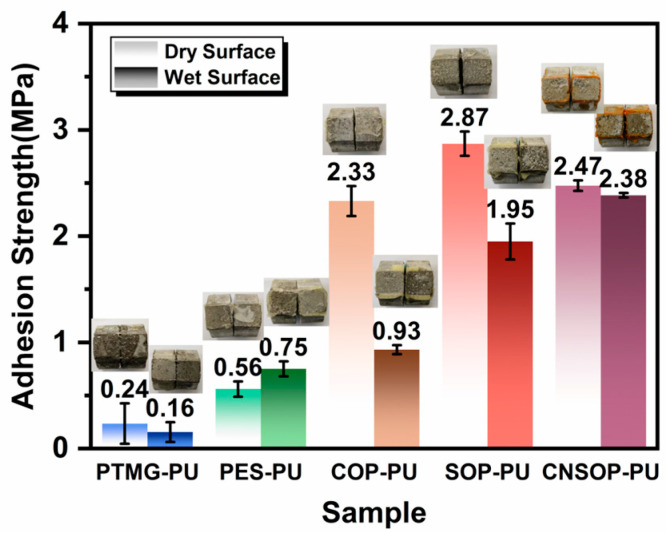
Adhesion strengths of samples on wet and dry mortar surfaces.

Figure 11 displays the adhesion strengths of polyurethane samples on dry and wet mortar substrates after a 7-day curing period, highlighting the performance of different polyurethanes for underwater applications. Adhesion tests on five sample sets show that bio-based polyurethanes generally exhibit better adhesion than conventional polyurethanes derived from polyether and polyester polyols. PTMG-PU exhibited the lowest adhesion strength due to its low crosslinking density, with dry and wet adhesion strengths of only 0.24 MPa and 0.16 MPa, respectively. PES-PU showed slightly higher adhesion on dry surfaces and a notable increase in wet adhesion strength, from 0.56 MPa to 0.75 MPa. This improvement can be attributed to the better wetting properties of polyester polyols on wet substrates, as shown in Figure 13, allowing for greater diffusion into the mortar’s capillary pores and increasing the effective bonding area. COP-PU showed the most significant reduction in adhesion strength, decreasing by 60%, from 2.33 MPa to 0.93 MPa. The slow reaction rate of castor oil-modified polyols, combined with side reactions during underwater curing that produce CO_2_ gas, reduces the effective bonding area. Additionally, some isocyanate groups undergo side reactions, leaving hydroxyl groups unreacted, which prevents full crosslinking of the polyol segments, further lowering adhesion strength. Consequently, the failure mode shifted from internal concrete failure to interface failure between the polyurethane and mortar. In contrast, SOP-PU and CNSOP-PU both exhibited adhesion failure primarily within the concrete matrix. SOP-PU had the highest dry adhesion strength at 2.87 MPa, but its wet adhesion strength decreased to 1.95 MPa, with a loss rate of 32%. As shown in Figure 13, the soy oil-modified polyol displayed limited spreading on the wet mortar surface, indicating poorer wettability. This reduced diffusion into the mortar’s capillary pores and diminished mechanical anchoring, leading to a decline in adhesion strength. CNSOP-PU exhibited the smallest loss in wet adhesion strength, with only a 3.6% decrease, yielding dry and wet adhesion strengths of 2.47 MPa and 2.38 MPa, respectively. In conclusion, bio-based polyurethanes generally show superior adhesion performance, with cashew nut shell oil-based polyurethane demonstrating the best wet adhesion, making it a promising candidate for underwater applications.

#### 3.1.6. Hydrophobicity of Different Polyol Types

During underwater casting, thoroughly mixed polyol and isocyanate components flow through water to reach the injection site. The interaction between the slurry and water molecules determines the extent of water bonding to the slurry [33]. Fewer water molecules binding to the droplet surface result in lower water content in the final reaction mixture, thereby minimizing side reactions between isocyanate and water. Additionally, the slurry’s wetting behavior and its spreading characteristics on both dry and wet substrates significantly influence the material’s performance. To assess the interaction between polyols and water during the casting process, the interfacial tension between polyol and water was measured, providing an estimate of water content in different systems under underwater casting conditions. Moreover, changes in the wetting diameter of slurry droplets on a mortar substrate over time were observed, with the wetting rates on both dry and wet surfaces compared. This approach provides insights into the hydrophobicity of samples and their wetting behavior on substrates.

**Figure 12:**
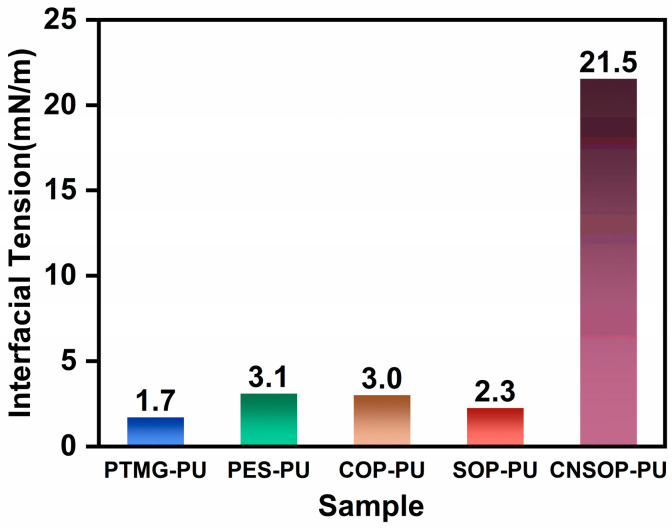
Interfacial tension between polyols and water.

The results presented in Figure 12 show that CNSOP, modified with cashew nut shell oil, exhibits significantly higher interfacial tension with water compared to the other samples. This suggests that the unique aromatic and long-chain structure of cashew nut shell oil-based polyols reduces the interaction with water molecules. As a result, these polyols are less likely to be wetted by water and are more readily able to penetrate water, making them more suitable for underwater casting or grouting applications. This property effectively reduces excess water entering into the reaction system, offering distinct advantages for underwater curing scenarios.

**Figure 13:**
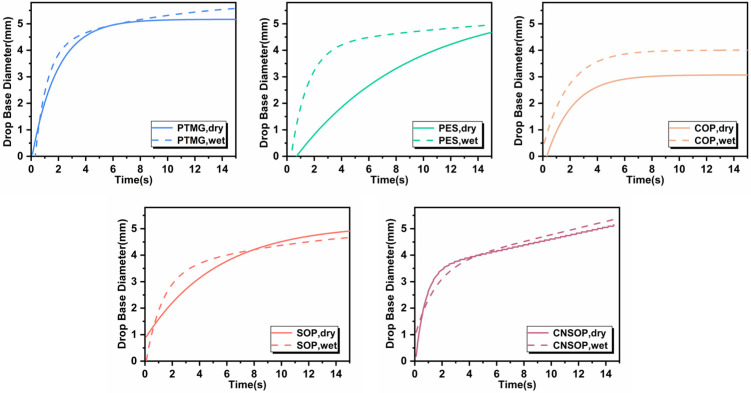
Droplet base diameter on dry and wet mortar surfaces over time.

Figure 13 shows the fitting curves of the base diameter of polyol droplets on dry and wet mortar surfaces over time. The curves are influenced not only by the slurry’s density but also by the interactions among the slurry, water molecules, and the mortar surface. Analysis of both the slope of the curves and the final spread diameter led to the following observations: Polyester polyol exhibited faster spreading on the wet surface, with a slightly larger final spread diameter on the wet substrate, likely due to capillary forces within the liquid layer. Polyester polyol showed better wettability on the wet substrate. Its ester group’s polarity facilitates stronger bonding with the concrete substrate, which is one reason for the increase in wet adhesion strength. Castor oil-modified polyol had a higher viscosity, leading to slower spreading on the dry surface. On the wet surface, the water film on the top layer of the slurry acted as a lubricant, promoting greater spreading and improving bonding with the substrate. Soy oil-modified polyol exhibited poorer wettability on wet surface compared to dry ones, which led to a larger decrease in wet adhesion strength. Cashew nut shell oil-modified polyol demonstrated a similar spreading behavior on both the dry and wet surfaces, indicating comparable wettability and minimal adhesion strength loss on wet substrates. From the interfacial tension and spreading behavior analysis, it can be concluded that, with the exception of the soy oil-modified polyol, all polyols exhibited better wettability on wet surfaces than on dry ones. Polyester polyol displayed the best wettability on wet surfaces, while cashew nut shell oil-modified polyols showed superior hydrophobicity with similar wettability on both dry and wet substrates. These properties make cashew nut shell oil-modified polyols particularly suitable for underwater applications, where consistent performance on both dry and wet surfaces is essential.

### 3.2. Effect of Polyol Types on the Mechanical Properties of Polyurethane

**Figure 14:**
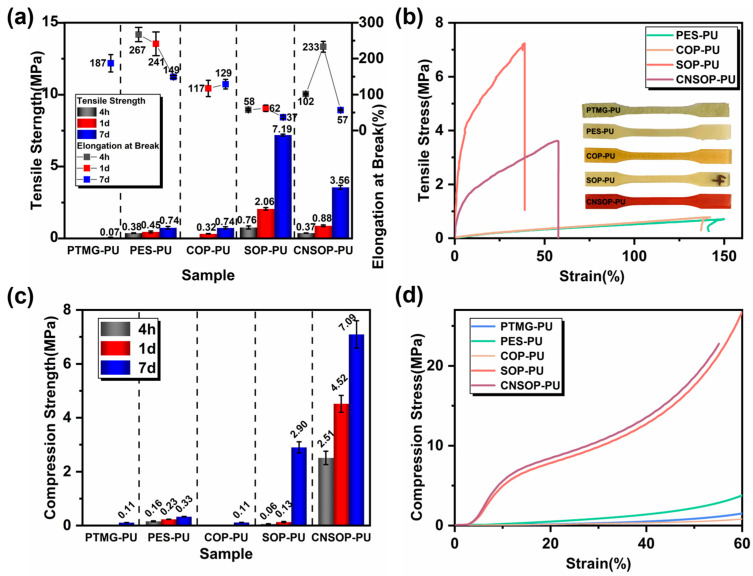
Mechanical properties and stress–strain curves of the samples. (**a**) Tensile properties of the samples; (**b**) tensile stress–strain curves; (**c**) compression properties of the samples; (**d**) compression stress–strain curves.

Figure 14 presents the tensile and compressive properties, along with the stress–strain curves, of five samples at different time intervals. PTMG-PU, due to its slow reaction rate at room temperature, showed only a weak strength after 7 days. PES-PU exhibited an increase in tensile strength over time, with a decrease in elongation at break. After 7 days, its tensile strength was 0.74 MPa, and the elongation at break was 149%, indicating continued curing and the formation of stronger urethane or urea bonds. This process likely increased hydrogen bonding and crosslinking density, leading to reduced elongation. These two samples displayed lower strength, likely due to their low functionality and crosslinking density. COP-PU, which contains ester bonds, exhibited slightly higher strength than PTMG-PU due to the increased cohesive energy and polarity of ester bonds, enabling more hydrogen bonding. However, due to its low reactivity, COP-PU formed poorly in the early stages, resulting in numerous bubble defects in the cured product, and its tensile strength after 7 days remained at 0.74 MPa. SOP-PU exhibited the highest tensile strength among all five polyol-based samples, reaching 7.18 MPa after 7 days. The superior mechanical properties of SOP-PU can be attributed to the high reactivity and functionality of the soybean oil-modified polyol, which facilitates greater crosslinking and stronger intermolecular interactions between hard segments. This results in enhanced mechanical properties and minimized side reactions during curing, reducing pore defects in the final product. The high tensile strength of SOP-PU may also stem from the larger molecular weight and complex branching of the soybean oil-modified polyol, which increases the number of polymer chains that break during stretching, further improving tensile strength. CNSOP-PU was soft and weak at 4 h but showed improved tensile strength and elongation at break after 1 day, indicating ongoing chain extension and crosslinking. After 7 days, the tensile strength reached 3.56 MPa, with an elongation at break of 57%. Compressive tests revealed that CNSOP-PU exhibited the highest compressive strength, reaching 7.09 MPa after 7 days, followed by SOP-PU at 2.90 MPa. The increased rigidity of the molecular chain in CNSOP-PU, due to the presence of phenyl rings in the cashew nut shell oil-based polyol, contributed to its superior mechanical performance.

## 4. Conclusions

1. To minimize the impact of side reactions between isocyanates and water in polyurethane systems for water-bearing environments, it is crucial to consider both gel time and slurry water solubility. Shorter gel times and better hydrophobicity lead to reduced side reactions, making the system more suitable for underwater applications. Among polyether, polyester, and bio-based polyols examined in this study, soybean oil- and cashew shell oil-modified polyols showed minimal side reactions, highlighting their potential for underwater construction. In contrast, polyether polyols were the least suitable, while castor oil-modified systems showed pronounced side reactions, particularly in underwater conditions.
2. Soybean oil-based and cashew nut shell oil-based polyurethanes demonstrated the best overall performance among the five polyols tested; their distinct mechanical properties, due to structural differences, allow for selection based on specific project requirements. PES-PU, with acceptable wet adhesion properties, may be suitable for interface bonding in low mechanical performance applications. SOP-PU, with higher tensile strength, could be considered for deformation joint filling or leakage seam repair, while CNSOP-PU, with higher compressive strength and hydrophobicity, may be suited for underwater stress-bearing applications, such as filling wet base surfaces or repairing tunnel gaps. Further adjustments to formulation may be required to optimize performance.
3. No clear correlation was observed between system temperature and the degree of side reactions. Further studies are needed to explore the effects of temperature on isocyanate-water side reactions and bubble dynamics during curing.

## Figures and Tables

**Table 1:** Detailed sample composition.

Sample	Polyol Type	Polyol Hydroxyl Value (mg KOH/g)	Polyol (wt%)	1,4-Butanediol (wt%)	Catalyst (wt%)	Isocyanate Component (wt%)
PTMG-PU	polyether polyol	173	60.08	3.17	2	34.75
PES-PU	polyester polyol	167	60.83	3.20	2	33.97
COP-PU	castor oil-modified polyol	195	57.95	3.05	2	37.00
SOP-PU	soybean oil-modified polyol	180	59.36	3.13	2	35.52
CNSOP-PU	cashew nut shell oil-modified polyol	175	59.72	3.15	2	35.14

## Data Availability

The original contributions presented in this study are included in the article. Further inquiries can be directed to the corresponding author.
